# Amikacin Therapy in Japanese Pediatric Patients: Narrative Review

**DOI:** 10.3390/ijerph19041972

**Published:** 2022-02-10

**Authors:** Hideo Kato, Yukihiro Hamada

**Affiliations:** 1 Department of Pharmacy, Mie University Hospital, Tsu 514-8507, Japan; 2 Department of Clinical Pharmaceutics, Division of Clinical Medical Science, Mie University Graduate School of Medicine, Tsu 514-8507, Japan; 3 Department of Pharmacy, Tokyo Women’s Medical University Hospital, Tokyo 162-8666, Japan; hamada.yukihiro@twmu.ac.jp

**Keywords:** pediatric patients, amikacin, pharmacokinetic/pharmacodynamic, Japanese

## Abstract

Children show a very wide range of physical development processes. These changes impact pharmacokinetic (PK) variability in pediatric patients. Most PK studies have been conducted on the Caucasian population. Therefore, whether current evidence of how developmental change affects PK and exposure-response relationships applies to Japanese pediatric patients remains unclear. This narrative review focuses on amikacin therapy in Japanese pediatric patients and shows the relationship between amikacin concentrations and efficacy/toxicity. Ten relevant articles were identified. Of these, nine articles were published in the 1980s. All studies reported a maximum concentration (Cmax) and minimum concentration (Cmin) of amikacin. Overall, articles reporting PK/pharmacodynamic (PD) indices and minimum inhibitory concentration (MIC) of isolated bacteria in Japanese pediatric patients is lacking, whereas all patients recovered from an infection state and showed negative cultures. Five of the included studies reported the association between Cmin and toxicity. The Cmin in three of four patients who developed toxicity was above 10 mg/L. This narrative review shows that further PK study of amikacin in Japanese pediatric patients is necessary. In particular, the pursuit of knowledge of Cmax/MIC ratio is vital. On the other hand, this review demonstrates that the optimal Cmin for Japanese pediatric patients is below 10 mg/L as a candidate concentration. However, it is noted that the number of patients who developed toxicity is very small.

## 1. Introduction

One after another, antibiotic-resistant bacteria have been emerging concomitantly with the development of new antibiotics [1]. It is expected that resistant bacteria will directly cause 10 million deaths by 2050 [2]. Appropriate administration is one of the important determinants of successful treatment and also prevents resistance to antibiotics. To date, numerous researchers all over the world have actively conducted pharmacokinetic/pharmacodynamic (PK/PD) studies to optimize antibiotic administration. However, most of the data are from studies conducted in the United States and Europe, and participants’ backgrounds such as standard body weight and metabolic enzymes can be different from those of Japanese people.

Amikacin is one of the aminoglycoside antibiotics that play a key role in treatment of serious, hospital-acquired infections with Gram-positive and Gram-negative pathogens [3]. The effect of amikacin is associated with the ratio of a maximum concentration (Cmax) to minimum inhibitory concentration (MIC; Cmax/MIC) as a PD parameter related to favorable clinical outcomes [4]. On the other hand, nephrotoxicity and ototoxicity are common adverse events reported from amikacin therapy and have been associated with high minimum concentration (Cmin) [3,5,6,7]. In clinical settings, an optimal dosage regimen of amikacin is complicated by its narrow therapeutic ranges. Moreover, the lack of studies in specific populations such as pediatric patients makes it much more difficult to optimize the dosing regimen for them.

Most antibiotics have been developed for adult participants, and dosing regimens of pediatric patients are extrapolated from adult data [8,9]. It should be noted that the expected effect cannot be obtained even when used at a dose per body weight, since pediatric patients include patients with a very wide range of physical development processes, from newborns to adolescents [10]. Moreover, it has been reported that these developmental changes greatly influence PK variability observed in pediatric patients [8,11]. In particular, approaches for dose selection for Japanese pediatric patients remain unclear, even with current evidence of how developmental growth affects PK and exposure–response relationships.

The aim of this narrative review is to illustrate the PK of amikacin in Japanese pediatric patients. Therefore, we performed a database search to identify all related English- and Japanese-language articles and abstracts reporting PK data in Japanese pediatric patients treated with amikacin.

## 2. Materials and Methods

### 2.1. Literature Serch

A literature review was conducted in accordance with the Preferred Reporting Items for Systematic Reviews and Meta-analysis (PRISMA) guidelines [12]. All literature updated in PubMed, Ichushi, and CINAHL until December 2021 was identified by an electronic search, using the following terms: “amikacin”, “child”, “neonate”, “infant”, and “Japanese”.

### 2.2. Study Selection

Articles that reported blood concentrations of amikacin in Japanese pediatric patients were included in this review. The types of publications that we considered included retrospective and prospective studies and case reports. The titles and abstracts of the articles found were reviewed and screened to identify eligible articles. Exclusion criteria were as follows: (i) review, (ii) duplicate publications, (iii) describing a study already included, (iv) non-clinical study, and (v) no data regarding amikacin concentrations.

### 2.3. Data Extraction

We extracted data on the following: study design, setting, number of patients, age, body weight, renal function, type of infection, bacteria, MIC of amikacin, amikacin regimen, treatment duration, Cmax, Cmin, Cmax/MIC, and clinical outcomes. The Cmax was defined as the highest concentration reached in patients [13]. We evaluated clinical effect, bacteriological effect, and adverse events as clinical outcomes. The clinical effect was defined as the absence of signs and/or symptoms. The bacteriological effect was defined as eradication of the pathogen. Adverse events were defined as incidences of nephrotoxicity or ototoxicity.

## 3. Results

**Figure 1:**
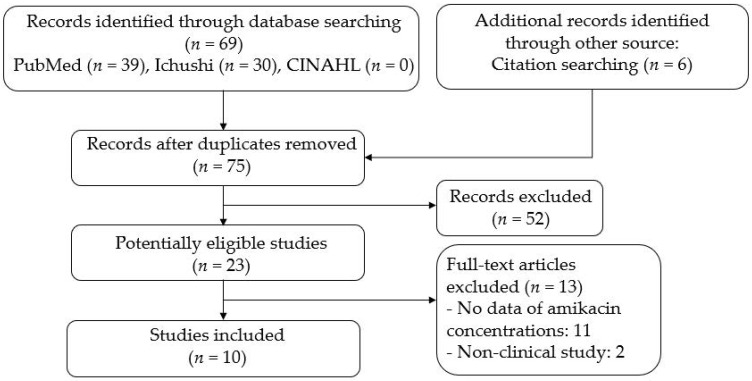
PRISMA flow chart for the selection of eligible studies.

Seventy-five potentially relevant articles were retrieved from the electronic databases, including six articles extracted using other methods such as citation searching. After screening the titles and abstracts of the remaining 75 articles, a full-text review of 23 articles was performed. Figure 1 presents the process and depicts the full list of reasons for exclusion. Finally, ten articles met the inclusion criteria [14,15,16,17,18,19,20,21,22,23]. Nine articles were published in 1980s, and only one was published in 2019. On the other hand, the number of potentially relevant articles excluding Japanese pediatric patients was 1787, and the articles were published during a broad duration of time, from 1975 to 2021.

### 3.1. Characteristics of Studies in Japanese Pediatric Patients Treated with Amikacin

Five studies were case reports [14,17,18,21,22], four were PK studies [15,16,19,20], and one was a retrospective study [23] (Table 1). All reports were single-center studies. The data of 102 Japanese pediatric patients was reported. Of these patients, ninety-three were infants [15,16,17,18,19,20,21,22,23] and nine were children [14,19]. Nine studies reported body weight of the included patients [14,15,16,17,18,19,20,22,23], while only one study reported renal function of the included patients [17]. Amikacin was administered as therapy for various infections: peritonitis, pneumonia, sepsis, urinary tract infection, skin and soft tissue infection, surgical site infection, and intraperitoneal infection [14,15,17,18,21,22,23]. The bacteria isolated in three studies were *Escherichia coli* and *Staphylococcus aureus* [14,17,21]. All bacteria were sensitive to amikacin (MIC of amikacin, 1.56 [14]; 0.78 [21]). No patient was treated with a combination with other antibiotics.

### 3.2. Overview of Amikacin Therapy and Clinical Outcomes in Japanese Pediatric Patients

An overview of amikacin therapy and clinical outcomes in the 10 studies is shown in Table 2. The single dose of amikacin was a mean of 5.1 mg/kg (range, 2.1–14.1 mg/kg). Amikacin was administered from one to three times per day, and five of the included studies reported an amikacin regimen of once-daily dosing [16,19,20,22,23]. Five studies reported the administration period [14,17,18,21,23], and the period was approximately 10 days. All the included studies reported the Cmax of amikacin, and only one study did not report the Cmin of amikacin [14]. The range of the Cmax value was 2.6 to 42.5 mg/L, and the range of the Cmin value was 0.3 to 28.4 mg/kg. On the other hand, Cmax/MICs ratio of four patients was calculated, since only two studies reported the MIC of isolated bacteria [14,21]. The mean Cmax/MIC ratio was 6.4 (2.9–11.7). All the patients in the five studies that reported a clinical effect fully recovered from their infection [14,17,18,21,23]. The clearance of isolated bacteria was confirmed with negative cultures of all the patients in three studies that reported a bacteriological effect [14,17,21]. Four studies reported no adverse events in pediatric patients treated with amikacin [14,17,18,21,23], and the most recent study reported that four patients had adverse effects caused by amikacin [23]. Of the four patients who experienced adverse events, three patients had a Cmin of over 10 mg/L.

Mean single-dose Cmax and Cmin values of amikacin in infants were 5.4 mg/kg (range, 2.1–14.1 mg/kg), 14.7 mg/L (5.9–29.1 mg/L), and 3.3 mg/L (0.8–7.9 mg/L). Mean single-dose values in children were 4.1 mg/kg (range, 3.0–5.2 mg/kg), 9.3 mg/L (7.1–11.5 mg/L), and 0.6 mg/L.

**Figure 2:**
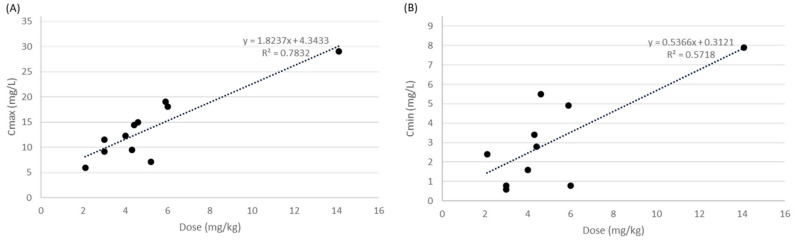
Mean single dose versus mean amikacin concentration in each study. (**A**), maximum concentration (Cmax); (**B**), minimum concentration (Cmin).

The relationships between one dose and amikacin concentrations (Cmax and Cmin) are shown in Figure 2. As one dose increased, a higher Cmax and Cmin was achieved (Cmax, R^2^ = 0.78, Figure 2A; Cmin, R^2^ = 0.57, Figure 2B).

## 4. Discussion

This narrative review shows that the PK of amikacin in Japanese pediatric patients has not yet been investigated well. In particular, we have shown that there is a lack of knowledge of the Cmax/MIC ratio. On the other hand, this review provides a candidate for the optimal Cmin for Japanese pediatric patients, since the association between the Cmin and adverse events is presented in several articles.

Recently, two reviews regarding the PK of antibiotics in pediatric patients have been published [24,25]. Neither of these reviews include any amikacin study performed in Japan. Although four PK studies and five case reports of Japanese pediatric patients treated with amikacin were found, these studies were written in Japanese. Therefore, our review is the first literature overview to summarize the available data on the PK of amikacin in Japanese pediatric patients and to provide evidence of amikacin therapy in Japan.

According to recent reviews [24,25], two cohort studies have reported the Cmax and Cmin of amikacin in pediatric patients [26,27]. The characteristics and overview of the PK studies included in the reviews are shown in Table 3 and Table 4. Bressolle F et al. [26] reported that 36 pediatric patients with various types of infections received doses ranging from 70 to 1500 mg. Mean Cmax and Cmin values were 40.7 and 0.97 mg/L, respectively. Two patients died, but their detailed information was not reported. Another study reported by Sherwin CMT et al. [27] included 73 pediatric patients who received a mean dosage of 16.4 mg/kg/day. Mean Cmax and Cmin values were 33.2 and 3.8 mg/L, respectively. However, clinical outcomes were not evaluated. Compared with these international data, the Cmin was within the concentration reported in Japanese pediatric patients. The Cmax of foreign patients was higher than that of Japanese patients, because foreign patients were administered higher doses than Japanese patients. However, a recent study of Japanese pediatric patients showed a similar Cmax associated with the administration of similar doses in international studies [23]. There was no detailed information regarding dead patients in the international studies. In the future, as further studies to optimize amikacin dosing regimens are needed, it should be noted that the optimal concentration of amikacin in Japanese pediatric patients is still unclear.

Two reviews [24,25] included no recent studies. On the other hand, our review included only one study published in 2019, whereas the others were studies published in the 1980s. Indeed, there were only three patients who were younger than 18 years old and for whom blood concentrations of amikacin were measured in our previous study [28]. That is, these three reviews indicate that studies that target pediatric patients treated with amikacin are very limited worldwide as well as those from in Japan.

Amikacin has concentration-dependent bactericidal activity [29]. In concentration-dependent antibiotics, both the Cmax/MIC and area under the concentration–time curve (AUC)/MIC ratios are main PK/PD parameters correlating with amikacin’s efficacy and toxicity. In the case of amikacin, the AUC/MIC ratio has been reported to be a better predictor of therapeutic efficacy than the Cmax/MIC ratio in animal studies, while the opposite has been reported in human clinical trials [30]. Generally, a Cmax/MIC ratio above 8 has been shown to result in a successful clinical outcome [31,32,33]. In our review, three of four patients for whom the Cmax/MIC ratio was calculated had a Cmax/MIC ratio below 8, whereas all the patients showed negative cultures and recovered from an infection state. This finding is consistent with our previous study on adult patients [28]. However, further studies are needed due to the very small sample of pediatric patients.

In addition, amikacin causes a post-antibiotic effect [29]. The post-antibiotic effect, which is defined as residual bactericidal activity continuing for some time after amikacin concentrations have reached values below the MIC, persists for up to 2–4 h after amikacin administration [33]. Therefore, there has been a debate regarding whether amikacin should be administered on a once-daily dosing or a multiple-daily dosing schedule. As results of a meta-analysis of amikacin administration for pediatric patients have indicated, there was no significant difference between the once-daily and the multiple-daily dosing in clinical failure rates and microbiologic failure rates [34]. These results are consistent with a meta-analysis of adult data [35]. Therefore, the antibiotic effects of amikacin may not differ between pediatric and adult patients. In our review, five of the included studies reported an amikacin regimen of three times per day, and the others reported once-daily dosing.

Amikacin exhibits a relatively high potential risk of nephrotoxicity and ototoxicity. The incidence of toxicity is difficult to establish because there are several factors such as type of infection, the duration of the treatment, and so on [33]. The Japanese practical guideline recommends a Cmin below 4 mg/L for adult patients [36]. Meanwhile, it is reported that the toxicity of amikacin in pediatric patients is associated with a Cmin above 10 mg/L [33,37]. Several available data [33,37,38,39] suggest that the risk of toxicity in pediatric patients is lower than in adults. In our review, three of four patients who developed toxicity showed a Cmin above 10 mg/L. Recently, some meta-analyses have been reported in order to establish the evidence of PK/PD [40,41]. There is one meta-analysis regarding the Cmin of amikacin for reducing the risk of toxicity in adults [42]. The results demonstrated that a Cmin above 10 mg/L increased the risk of toxicity caused by amikacin. Therefore, tolerability of amikacin in pediatric patients may be similar to that in adult patients.

Several limitations should be noted. First, all the studies are single-center, retrospective studies, and nine of them were published in the 1980s. Second, we were unable to reveal the optimal concentration or PK/PD parameter because there are limited data on the outcomes and MIC. Finally, the nine studies have been written in Japanese. However, the current problems of amikacin therapy in Japanese pediatric patients can be recognized though this review.

## 5. Conclusions

In conclusion, this narrative literature review showed an overview of amikacin therapy in Japanese pediatric patients in spite of a very limited number of articles and provided challenges to be addressed and new findings. That is, we need to perform animal studies or hollow fiber infection studies in order to clarify the Cmax/MIC ratio of amikacin, and subsequently conduct clinical studies in order to confirm the Cmax/MIC ratio gained from non-clinical studies. In terms of the Cmin, further large-scale studies are necessary to obtain more robust evidence. This review aims to inspire Japanese researchers to close gaps to optimize amikacin treatment, as the data for Japanese pediatric patients available now are lacking.

## Figures and Tables

**Table 1:** Summary of all included studies on Japanese pediatric patients treated with amikacin.

Study	Study Design	Setting	No of Patients	Age	Body Weight (kg)	Renal Function	Type of Infection	Bacteria	MIC of AMK (mg/L)
Nakamura T, 1982	Case report	Single- center	3	9 years (8–15 years)	38 (29–57)	NR	Peritonitis	*E. coli*	1.56
Hashira S, 1987	PK study	Single- center	21	5 days (0–25 days)	2.8 (0.6–3.9)	NR	Pneumonia (*n* = 7); sepsis (*n* = 3); others (*n* = 11)	NR	NR
Iwai N, 1987	PK study	Single- center	24	25.5 days (8–365 days)	3.4 (1.9–11.5)	NR	NR	NR	NR
Kuroki S, 1987	Case report	Single- center	2	45.0 days (26–64 days)	4.6 (3.8–5.3)	Scr 0.45 (0.40–0.50)	UTI	*E. coli*	Sensitivity against AMK
Masumi R, 1987	Case report	Single- center	2	0.5 days (0–1 days)	2.7 (2.4–2.9)	NR	Pneumonia	NR	NR
Motohiro T, 1987	PK study	Single- center	15: children, *n* = 6; neonate, *n* = 9	Children, 9.3 years (7–11 years); neonate, 12 days (4–18 days)	Children, 25.0 (21.1–31.9); neonate, 2.1 (1.4–3.3)	NR	NR	NR	NR
Nanri S, 1987	PK study	Single- center	13	2.9 days (0–11 days)	2.6 (1.9–4.1)	NR	NR	NR	NR
Nishimura T, 1987	Case report	Single- center	1	13 days	NR	NR	SSTI	*S. aureus*	0.78
Yura J, 1987	Case report	Single- center	1	18 days	1.7	NR	SSI	NR	NR
Endo A, 2019	Retrospective study	Single- center	20	GA, 30 ± 5.1 weeks	1.3 ± 0.8	NR	Sepsis; pneumonia; intraperitoneal infection	NR	NR

The values represent the following: mean (minimum–maximum); mean ± SD. AMK, amikacin; MIC, minimum inhibitory concentration; NR, not reported; PK, pharmacokinetic; Scr, serum creatinine; SD, standard deviation; SSI, surgical site infection; SSTI, skin and soft tissue infection; UTI, urinary tract infection.

**Table 2:** Overview of all included studies performed on Japanese pediatric patients.

Study	Regimen	Treatment Duration (day)	Sampling Time after AMK Administration	Cmax (mg/L)	Cmin (mg/L)	Cmax/MIC	Clinical Effect	Bacteriological Effect	Adverse Event
Nakamura T, 1982	5.3 mg/kg (3.5–6.9 mg/kg) every 12 h	5 (4–9)	Cmax: 1.5 h Cmix: NR	7.0 (4.6–9.8)	NR	4.5 (2.9–6.3)	All patients cured.	Bacteriological cure rate was 100%.	None
Hashira S, 1987	5.0 mg/kg (2.0–7.5 mg/kg) every 12 h	NR	Cmax: 0.5–1 h Cmin: 12 h	14.4 (4.5–37.7)	1.9 (0.6–9.3)	NR	NR	NR	NR
Iwai N, 1987	3.0 mg/kg (1.4–6.0 mg/kg) every 24 h	NR	Cmax: 0.5–1 h Cmin: 8 h	8.7 (2.6–28.5)	1.3 (0.8–5.2)	NR	NR	NR	NR
Kuroki S, 1987	5.9 mg/kg (5.7–6.1 mg/kg) every 8 h	7 (7–7)	Cmax: 0.5–1 h Cmin: 8 h	19.0 (18.0–20.0)	4.9 (3.8–6)	NR	All patients cured.	Bacteriological cure rate was 100%.	None
Masumi R, 1987	2.1 mg/kg (1.6–2.5 mg/kg) every 12 h	3 (3–3)	Cmax: 0.5–1 h Cmin: 8–12 h	5.9 (3.8–8.0)	2.4 (2.0–2.7)	NR	All patients cured.	NR	None
Motohiro T, 1987	Children, 3.0 mg/kg (2.0–4.0 mg/kg) every 24 h; neonate, 4.3 mg/kg (3.0–6.0 mg/kg) every 24 h	NR	Cmax: 0.5–1 h Cmin: 6 h	Children, 11.5 (8.2–13.9); neonate, 9.5 (6.1–16.2)	Children, 0.6 (0.3–1.1); neonate, 3.4 (1.7–6.6)	NR	NR	NR	NR
Nanri S, 1987	4.6 mg/kg (3.0–6.0 mg/kg) every 24 h	NR	Cmax: 0.5–1 h Cmin: 6 h	15.0 (6.3–26.3)	5.5 (2.1–10.4)	NR	NR	NR	NR
Nishimura T, 1987	3 mg/kg every 8 h	7	Cmax: 0.5 h Cmin: 8 h	9.1	0.8	11.7	Cured	The culture on day 5 showed negative.	None
Yura J, 1987	6.0 mg/kg every 24 h	NR	Cmax: 0.5 h Cmin: 8 h	18.1	0.8	NR	NR	NR	NR
Endo A, 2019	14.1 ± 2.6 mg/kg every 24 h	10.1 ± 4.1	Cmax: 1–1.5 h Cmin: 23–24 h	29.1 (19.4–42.5)	7.9 (1.8–28.4)	NR	All patients cured.	NR	20% (4/20, 3 patients of them had trough concentrations ≥ 10 mg/L)

The values represent the mean (minimum–maximum). AMK, amikacin; h, hour; MIC, minimum inhibitory concentration; NR, not reported.

**Table 3:** Summary of two PK studies included in recent reviews.

Study	Study Design	Setting	No of Patients	Age	Body Weight (kg)	Renal Function	Type of Infection	Bacteria	MIC of AMK (mg/L)
Bressolle F, 1996	PK study	Single- center	36	5.7 years (6 months–15 years)	20.4 ± 13.6	Scr 49.1 ± 17.5 μmol/L	Pneumonia (*n* = 4); UTI (*n* = 11); bone and joint (*n* = 5); sepsis (*n* = 2); SSTI (*n* = 5); gastrointestinal tract (*n* = 6); Others (*n* = 3)	*E. coli* (*n* = 10); *P. aeruginosa* (*n* = 2); *M. catarrhalis* (*n* = 1); *Klebsiella* sp (*n* = 2); *S. aureus* (*n* = 5); *S. epidermidis* (*n* = 1); *Streptococcus* sp (*n* = 3); *Salmonella* sp (*n* = 5); *E. cloacae* (*n* = 1)	NR
Sherwin CMT, 2014	PK study	Single- center	73	Median 4.5 years (0.6–17 years)	Median 20 (8–90)	NR	Burn	NR	NR

The values represent the following: mean (minimum–maximum); mean ± SD. AMK, amikacin; MIC, minimum inhibitory concentration; NR, not reported; PK, pharmacokinetic; Scr, serum creatinine; SD, standard deviation; SSTI, skin and soft tissue infection; UTI, urinary tract infection.

**Table 4:** Overview of two PK studies included in recent reviews.

Study	Regimen	Treatment Duration (day)	Cmax (mg/L)	Cmin (mg/L)	Cmax/MIC	Clinical Effect	Bacteriological Effect	Adverse Event
Bressolle F, 1996	70–1500 mg every 24 h	8.8 ± 3.0	40.7 ± 15.8	0.97 ± 0.66	NR	Two patients died.	NR	NR
Sherwin CMT, 2014	16.4 ± 3.9 mg/kg/day (4.9–22.3 mg/kg/day)	NR	33.2 ± 9.4	3.8 ± 4.6	NR	NR	NR	NR

The values represent the following: mean (minimum–maximum); mean ± SD; h, hour; MIC, minimum inhibitory concentration; NR, not reported; SD, standard deviation.

## Data Availability

All data are applicable in the paper.
